# Subtype specific genetic associations for juvenile idiopathic arthritis: *ERAP1 *with the enthesitis related arthritis subtype and *IL23R *with juvenile psoriatic arthritis

**DOI:** 10.1186/ar3235

**Published:** 2011-01-31

**Authors:** Anne Hinks, Paul Martin, Edward Flynn, Steve Eyre, Jon Packham, Anne Barton, Jane Worthington, Wendy Thomson

**Affiliations:** 1 Arthritis Research UK Epidemiology Unit, Manchester Academic Health Science Centre, Stopford Building, Oxford Road, The University of Manchester, Manchester, M13 9PT, UK; 2 Haywood Hospital, University Hospital of North Staffordshire, High Lane, Stoke on Trent, Staffordshire, ST4 7LN, UK

## Abstract

**Introduction:**

Juvenile idiopathic arthritis (JIA) is an umbrella term for all chronic childhood arthropathies and can be divided into seven subtypes. It includes the enthesitis related arthritis (ERA) subtype which displays symptoms similar to ankylosing spondylitis (AS) and juvenile-onset psoriatic arthritis which has similarities to psoriatic arthritis (PsA) and psoriasis (Ps). We, therefore, hypothesized that two well-established susceptibility loci for AS and Ps, *ERAP1 *and *IL23R*, could also confer susceptibility to these JIA subtypes.

**Methods:**

Single nucleotide polymorphisms (SNPs) in *ERAP1 *(rs30187) and *IL23R *(rs11209026) were genotyped in JIA cases (n = 1,054) and healthy controls (n = 5,200). Genotype frequencies were compared between all JIA cases and controls using the Cochrane-Armitage trend test implemented in PLINK. Stratified analysis by ILAR subtype was performed.

**Results:**

The ERA subtype showed strong association with *ERAP1 *SNP (*P *trend = 0.005). The *IL23R *SNP showed significant association in the PsA subtype (*P *trend = 0.04). The SNPs were not associated with JIA overall or with any other subtype.

**Conclusions:**

We present evidence for subtype specific association of the *ERAP1 *gene with ERA JIA and the *IL23R *gene with juvenile-onset PsA. The findings will require validation in independent JIA datasets. These results suggest distinct pathogenic pathways in these subtypes.

## Introduction

There is now strong evidence supporting the hypothesis of common autoimmune genetic susceptibility loci [1]. Ankylosing spondylitis (AS), psoriasis (Ps) and psoriatic arthritis (PsA) are a group of autoimmune diseases which share some clinical features and some overlapping susceptibility loci such as endoplasmic reticulum aminopeptidase 1 (*ERAP1*) (formerly known as *ARTS1*) [2,3] and the interleukin 23 receptor (*IL23R*) [2,4,5].

Juvenile idiopathic arthritis (JIA) is an umbrella term for all chronic childhood arthropathies and can be classified into seven subtypes on the basis of features present in the first six months of disease [6]. It includes the enthesitis related arthritis (ERA) subtype, which displays symptoms similar to AS and juvenile onset psoriatic arthritis, which has similarities to psoriatic arthritis and psoriasis. We have, therefore, hypothesized that the two well-established susceptibility loci, *ERAP1 *and *IL23R*, also confer susceptibility to these JIA subtypes. To this end the most associated single nucleotide polymorphism (SNP) within each of these genes has been selected for genotyping across all JIA and also analysed stratified by ILAR subtype.

## Materials and methods

### Subjects

For the analysis of the *ERAP1 *SNP, DNA was available for 1,054 UK Caucasian JIA patients (332 males, 715 females) as previously described [7]. JIA cases were classified according to ILAR criteria [6]. The numbers genotyped per ILAR subgroup were: Systemic onset (n = 164), persistent oligoarthritis (n = 297), extended oligoarthritis (n = 147), rheumatoid factor (RF) negative polyarticular JIA (n = 215), RF positive polyarticular JIA (n = 68), enthesitis related JIA (n = 63), psoriatic JIA (n = 76) and unclassified (n = 24). For the analysis of the *IL23R *SNP, there were an additional 190 JIA cases available, making a total of 1,244 JIA cases. The numbers genotyped per ILAR subgroup were: Systemic onset (n = 179), persistent oligoarthritis (n = 380), extended oligoarthritis (n = 159), rheumatoid factor (RF) negative polyarticular JIA (n = 259), RF positive polyarticular JIA (n = 76), enthesitis related JIA (n = 74), psoriatic JIA (n = 93) and unclassified (n = 24). All individuals were recruited with ethical approval and provided informed consent (North-West Multi-Centre Research Ethics Committee (MREC 99/8/84) and the University of Manchester Committee on the Ethics of Research on Human Beings).

The control genotyping data were extracted from the Wellcome Trust case control consortium 2 (WTCCC2) European Genome-phenome Archive (EGA) website [8], both SNPs had been genotyped on the Illumina platform (n = 5,200).

### SNP selection

SNPs in *IL23R *(rs11209026) and *ERAP1 *(rs30187) previously associated with AS and Ps were selected for genotyping.

### Genotyping

All SNPs were genotyped, in UK JIA cases, using the Sequenom iPlex^® ^MassARRAY platform according to manufacturer's instructions (Sequenom, San Diego, CA, USA [9]). A 90% sample quality control rate and 90% genotyping success rate was imposed on the analysis.

### Statistical analysis

Power calculations were performed using QUANTO [10] based on the effect sizes reported in previous studies of these SNPs in AS [2,11]. Calculations assumed a log-additive model and an alpha value of 0.05. Genotype and allele frequencies were compared between cases with JIA and controls using STATA version 9 SE (StataCorp, College Station, Texas, USA) and PLINK [12]. Associations were expressed as odds ratios (ORs) and 95% confidence intervals (CIs). There was a strong prior hypothesis that these SNPs would be associated with the ERA and PsA subtypes of JIA; therefore, we performed stratified analysis by ILAR subtype.

## Results

SNPs were in Hardy-Weinberg equilibrium (*P *> 0.05) in the control cohort.

Neither rs30187 in *ERAP1 *nor rs11209026 in *IL23R *were significantly associated with the total JIA dataset (*P *trend = 0.73, OR = 1.03 95% CI 0.92 to 1.14 and *P *trend = 0.46, OR = 0.93 95% CI 0.78 to 1.12, respectively).

After stratification by ILAR subtype (Table 1) the ERA subtype showed strong association with the *ERAP1 *SNP (*P *trend = 0.005, OR = 1.69 95% CI 1.17 to 2.44). For the *IL23R *SNP there was significant association in the psoriatic arthritis subtype (*P *trend = 0.04, OR = 0.4 95% CI 0.16 to 0.98) and a trend towards association in the enthesitis related subtype (*P *trend = 0.15, OR = 0.52 95% CI 0.21 to 1.28). For both SNPs the association is in the same direction as in the original studies.

**Table 1:** Association analysis of *IL23R *(rs11209026) and *ERAP1 *(rs30187) SNPs in all JIA and stratified by subtype

	IL23R (rs11209026)				ERAP1 (rs30187)			
		
ILAR subtype	MAF Cases	MAF Controls	*P *Trend	OR 95% CI	MAF Cases	MAF Controls	*P *Trend	OR 95% CI
All JIA	0.06	0.07	0.46	0.93 (0.78 to 1.12	0.34	0.33	0.65	1.02 (0.92 to 1.13
Systemic	0.08	0.07	0.44	1.17 (0.78 to 1.76)	0.35	0.33	0.56	1.07 (0.85 to 1.36)
Persistent oligoarthritis	0.07	0.07	0.85	1.03 (0.77 to 1.39)	0.31	0.33	0.31	0.91 (0.76 to 1.09)
Extended oligoarthritis	0.07	0.07	0.65	1.11 (0.71 to 1.72)	0.34	0.33	0.84	1.03 (0.80 to 1.32)
RF negative polyarthritis	0.06	0.07	0.5	0.88 (0.6 to 1.29)	0.30	0.33	0.19	0.86 (0.70 to 1.07)
RF positive oligoarthritis	0.07	0.07	0.93	0.97 (0.49 to 1.91)	0.39	0.33	0.22	1.26 (0.87 to 1.82)
Enthesitis related arthritis	0.04	0.07	0.15	0.52 (0.21 to 1.28)	**0.46**	**0.33**	**0.005**	**1.69** **(1.178 to 2.44)**
Psoriatic arthritis	**0.03**	**0.07**	**0.04**	**0.4** **(0.16 to 0.98)**	0.38	0.33	0.21	1.24 (0.88 to 1.74)

CI, confidence interval; ERAP1, endoplasmic reticulum aminopeptidase 1; IL23R, interleukin 23 receptor; JIA, juvenile idiopathic arthritis; MAF, minor allele frequency; OR, odds ratio.

## Discussion

Investigating the genetic susceptibility loci identified for clinically related autoimmune diseases has been a very successful strategy in the search for novel JIA susceptibility loci [7]. Using this approach in this study we have identified subtype specific effects in JIA.

We found strong association of the ERA subtype with the *ERAP1 *SNP. There was no significant association with any other subtype. However, it should be noted that the individual subtypes had between 16 and 54% power to detect an association. The *ERAP1 *variant, rs30187, is a non-synonymous SNP, K528R, located in exon 6 of the gene previously associated with AS [2], with Ps [3] and PsA (personal communication, Anne Barton), and also with type 1 diabetes [13]. In a recent fine-mapping study, multiple SNPs across the region were associated with AS and they were not able to identify the causal marker from the genetic data due to the high LD across the region. Imputation analysis in this dataset found strong associations for imputed SNPs, including some SNPs in regulatory regions which may be good candidates for the causal SNP. Further studies on how these SNPs affect expression of ERAP1 are now required [11].

*ERAP1 *encodes a multifunctional aminopeptidase, but its role in the pathogenesis in any of the associated diseases has yet to be determined (reviewed in [14]). It may play a role in trimming peptides, in the endoplasmic reticulum, for binding to HLA class I molecules where they are transported to the cell surface for presentation to T cells. Alternatively it may be important through its function in cleaving pro-inflammatory cytokine receptors, such as tumour necrosis factor receptor 1 (TNFR1) to generate soluble TNFR1. It is also thought to play a role in the cleavage of interleukin 1 receptor 2 (IL1R2) and interleukin 6 receptor alpha (IL6Rα), leading to increased soluble IL1R2 and IL6Rα.

The *IL23R *SNP showed significant association in the PsA subtype and a trend towards association in the ERA subtype, with similar effect sizes in both subtypes, suggesting that the lack of significant association in the ERA subtype of JIA may just be due to a lack of power (this subtype had 37% power to detect an association). *IL23R *shows association not only with AS, Ps, PsA [2,4,5], but also with inflammatory bowel disease [15] and paediatric Crohn's disease [16]. The SNP rs11209026 appears to be the primary associated polymorphism [17]. It is a non-synonymous SNP, Q381R, located in exon 5 of the gene. In all cases the minor allele is associated with protection for developing the disease. The association of *IL23R *with these conditions suggests that the T Helper 17 (TH17) pathway may be involved in disease pathogenesis. IL23R is also expressed on other immunological cell types such as macrophages, natural killer cells and microglia and it is not yet determined which cell type is affected by the *IL23R *variant. Therapeutic approaches to inhibiting TH17 cells are now being investigated that could be useful in the treatment of juvenile-onset PsA and the ERA subtype of JIA.

To date it has been very difficult to identify ILAR subtype specific effects in JIA due to small sample sizes and the inevitable loss of power that this entails as well as the issues of multiple testing. In this case there was a strong prior hypothesis that *ERAP1 *and *IL23R *confer susceptibility to particular subtypes. However, the results will require validation in independent datasets. The lack of association across the other subtypes will also require further investigation. For the *ERAP1 *SNP we had between 16 and 54% power to detect an association across the subtypes and for the *IL23R *SNP we had between 37 and 95% power to detect an association. The search for subtype specific effects in JIA is imperative because it will aid in the refinement of the JIA classification, potentially lead to a better understanding of subtype specific disease pathogenesis, and ultimately to the development of more targeted therapies for the specific subtypes of JIA. It may also be relevant for evaluating prognosis. Not all children with ERA will go on to have the complete AS phenotype and it may be that carriage of these risk alleles predict progression to axial involvement and eventual ankylosis [18].

## Conclusions

We present evidence for subtype specific association of the *IL23R *gene with juvenile-onset PsA and *ERAP1 *gene with the ERA subtype of JIA. These results suggest distinct pathogenic pathways in these subtypes.

## Abbreviations

AS: ankylosing spondylitis; CI: Confidence interval; EGA: European Genome-phenome Archive; ERA: enthesitis related arthritis; ERAP1: endoplasmic reticulum aminopeptidase 1; HWE: Hardy-Weinberg equilibrium; IL1R2: interleukin 1 receptor 2; IL6Rα: interleukin 6 receptor alpha; IL23R: interleukin 23 receptor; ILAR: International League of Associations for Rheumatology; JIA: juvenile idiopathic arthritis; OR: odds ratio; PsA: psoriatic arthritis; Ps: psoriasis; SNP: single nucleotide polymorphism; TH17: T helper 17; TNFR1: tumour necrosis factor receptor 1; WTCCC2: Wellcome Trust Case Control Consortium 2.

## Competing interests

The authors declare that they have no competing interests.

## Authors' contributions

AH, WT, SE, JW and AB conceived the study. PM and EF performed the genotyping; AH undertook the statistical analysis. JP, CAPs and the BSPAR study group consortia provided samples. AH, WT and AB drafted the manuscript and all authors contributed to and approved the final version.
